# Supplementary material for: Trait-associated noncoding variant regions affect TBX3 regulation and cardiac conduction
Source: eLife. 2020 Jul 16;9:e56697. doi: 10.7554/eLife.56697 (PMC7365664; doi:10.7554/eLife.56697)
Supplement: Supplementary file 1. — Supplementary Table 1. Transcription factor binding motif enrichment in genomic regions accessible in E14.5 atrioventricular junctions. Transcription factor binding motif enrichment in 67 genomic regions accessible in E14.5 atrioventricular junctions (AVJ) or E14.5 ventricles. Enrichment was compared to enrichment in a number of random control sequences. Enrichment is depicted as total number of regions with the motif and the percentage of the total number of tested regions. P-values depict statistical significance of enrichment. Supplementary Table 2. Transcription factor binding motif enrichment in active STARR-regions. Transcription factor binding motif enrichment in genomic regions within both mouse (A) and human (B) TBX3 locus that respond to Smad/Gata (SG4) or Tcf (Wnt) factors as demonstrated by STARR-seq. Enrichment of binding motifs for Smad/Gata- and Tcf-factors is depicted. Enrichment was compared to enrichment in a number of random control sequences. Enrichment is depicted as total number of regions with the motif and the percentage of the total number of tested regions. P-values depict statistical significance of enrichment. Supplementary Table 3. Murine RE candidates within VR1 and VR2. Murine RE candidates selected based on accessible chromatin in AV junction cardiomyocytes (ATAC_AVJ), EMERGE prediction, or mSTARR_SG4/Wnt activity. Overlap is depicted of candidate regions with ChIP-seq peaks from publicly available datasets for Gata4 (differentiated cardiomyocytes) (Luna-Zurita et al., 2016), Hand2 (E10.5 heart) (Laurent et al., 2017) and H3K27ac (E11.5 heart) (Nord et al., 2013). Supplementary Table 4. Human RE candidates within VR1 and VR2. Human RE candidates selected based on mouse RE homology (mm9 liftover), EMERGE prediction, or hSTARR_SG4/Wnt activity. Regions overlapping GWAS SNPs (SNP id, trait association and respective p-value are listed) were selected for further analysis. Supplementary Table 5. TALEN/CRISPR target sequences for deletion o [file elife-56697-supp1.docx]

**SUPPLEMENTARY FILE 1**

**Supplementary Table 1.** Transcription factor binding motif enrichment in genomic regions accessible in E14.5 atrioventricular junctions

|  | **Motif enrichment** |  |  |  |
| --- | --- | --- | --- | --- |
| **Motif name** | **AVJ (67) vs Ctrl (273)** | **P-value** | **Ventricle (67) vs Ctrl (248)** | **P-value** |
| Smad3 | 50 (74.6%); 104 (38.0%) | 10^-7^ | 36 (53.7%); 88 (35.2%) | 10^-2^ |
| Hand2 | 12 (17.9%); 8 (2.9%) | 10^-4^ | 9 (13.4%); 16 (6.5%) | 10^-1^ |
| Smad2 | 40 (59.7%); 89 (32.4%) | 10^-4^ | 23 (34.3%); 69 (27.6%) | 1 |
| Gata6 | 19 (28.4%); 24 (8.8%) | 10^-4^ | 112 (74.7%); 31 (12.3%) | 10^-1^ |
| Tcf4 | 11 (16.4%); 9 (3.1%) | 10^-3^ | 6 (9.0%); 8.9 (3.6%) | 10^-1^ |
| Smad4 | 36 (53.7%); 83 (30.5%) | 10^-3^ | 21 (31.3%); 70 (28.1%) | 10 |
| Sox9 | 21 (31.3%); 45 (16.6%) | 10^-2^ | 14 (20.9%); 24 (9.7%) | 10^-1^ |
| Tcf3 | 6 (9.0%); 5 (1.8%) | 10^-2^ | 3 (4.5%); 9 (3.7%) | 10 |
| Tbx20 | 8 (11.9%); 9 (3.2%) | 10^-2^ | 7 (10.5%); 2.5 (1.0%) | 10^-3^ |
| Gata4 | 21 (31.3%); 44 (16.3%) | 10^-2^ | 15 (22.4%); 35 (14.2%) | 10^-1^ |
| MyoD | 12 (17.9%); 18 (6.7%) | 10^-2^ | 13 (19.4%); 19 (7.78%) | 10^-2^ |

Transcription factor binding motif enrichment in 67 genomic regions accessible in E14.5 atrioventricular junctions (AVJ) or E14.5 ventricles. Enrichment was compared to enrichment in a number of random control sequences. Enrichment is depicted as total number of regions with the motif and the percentage of the total number of tested regions. P-values depict statistical significance of enrichment.

**Supplementary Table 2.** Transcription factor binding motif enrichment in active STARR-regions

**A**

|  | **Motif enrichment (mouse)** | | |  |
| --- | --- | --- | --- | --- |
| **Motif name** | **SG4 (216) vs Ctrl (837)** | **P-value** | **Wnt (257) vs Ctrl (987)** | **P-value** |
| Smad3 | 168 (77.8%); 481 (57.4%) | 10^-7^ | 190 (75.1%); 551 (55.9%) | 10^-8^ |
| Smad4 | 141 (65.3%); 354 (42.3%) | 10^-8^ | 152 (60.1%); 369 (37,4%) | 10^-10^ |
| Smad2 | 128 (59.3%); 358 (42.8%) | 10^-4^ | 138 (54.6%); 381 (38.6%) | 10^-5^ |
| Tbox:Smad | 22 (10.2%); 30 (3.5%) | 10^-3^ | 21 (8.3%); 60 (6.1%) | 1 |
| Gata6 | 94 (43.5%); 328 (39.2%) | 1 | 10 (3.9%); 64 (6.5%) | 1 |
| Gata4 | 71 (32.3%); 244 (29.2%) | 1 | 82 (32.4%); 292 (29.6%) | 1 |
|  |  |  |  |  |
| Tcf4 | 50 (23.1%); 101 (12.1%) | 10^-4^ | 51 (20.1%); 90 (9.1%) | 10^-5^ |
| Tcf3 | 24 (11.1%); 61 (7.3%) | 10^-1^ | 31 (12.2%); 53 (5.4%) | 10^-3^ |
| Tcf12 | 70 (32.4%); 118 (41.1%) | 10^-8^ | 112 (44.3%); 131 (13.3%) | 10^-24^ |
| Tcf21 | 74 (34.3%); 130 (15.5%) | 10^-1^ | 115 (45.5%); 147 (14.8%) | 10^-22^ |

**B**

|  | **Motif enrichment (human)** | | | |
| --- | --- | --- | --- | --- |
| **Motif name** | **SG4 (216) vs Ctrl (537)** | **P-value** | **Wnt (257) vs Ctrl (591)** | **P-value** |
| Smad3 | 112 (74.7%); 278 (51.8%) | 10^-6^ | 104 (69.3%); 1276 (46.8%) | 10^-6^ |
| Smad4 | 79 (52.7%); 197 (36.7%) | 10^-3^ | 71 (47.3%); 160 (27.1%) | 10^-5^ |
| Smad2 | 84 (56.0%); 196 (36.5%) | 10^-4^ | 74 (49.3%); 168 (28.5%) | 10^-5^ |
| Tbox:Smad | 15 (10.0%); 12 (2.3%) | 10^-4^ | 10 (6.7%); 18 (3.0%) | 10^-1^ |
| Gata6 | 75 (50.0%); 197 (36.7%) | 10^-2^ | 59 (39.3%); 209 (35.3%) | 1 |
| Gata4 | 80 (53.3%); 218 (40.1%) | 10^-2^ | 65 (43.3%); 236 (40.0%) | 1 |
|  |  |  |  |  |
| Tcf4 | 39 (26.0%); 79 (14.8%) | 10^-2^ | 40 (26.7%); 67 (11.4%) | 10^-5^ |
| Tcf3 | 22 (14.7%); 45 (8.3%) | 10^-1^ | 27 (18.0%); 35 (6.0%) | 10^-4^ |
| Tcf12 | 41 (27.3%); 59 (11.0%) | 10^-5^ | 58 (38.7%); 51 (8.6%) | 10^-16^ |
| Tcf21 | 48 (32.0%); 72 (13.4%) | 10^-6^ | 57 (38.0%); 75 (12.8%) | 10^-10^ |

Transcription factor binding motif enrichment in genomic regions within both mouse (A) and human (B) *TBX3* locus that respond to Smad/Gata (SG4) or Tcf (Wnt) factors as demonstrated by STARR-seq. Enrichment of binding motifs for Smad/Gata- and Tcf-factors is depicted. Enrichment was compared to enrichment in a number of random control sequences. Enrichment is depicted as total number of regions with the motif and the percentage of the total number of tested regions. P-values depict statistical significance of enrichment.

**Supplementary Table 3.** Murine RE candidates within VR1 and VR2.

|  | **ATAC** |  |  | ***m*STARR** | |  | **ChIP** | | |
| --- | --- | --- | --- | --- | --- | --- | --- | --- | --- |
|  | **AVJ** | **EMERGE** |  | **SG4** | **Wnt** |  | **Gata4** | **Hand2** | **H3K27ac** |
| mRE1 | + |  |  |  |  |  |  |  |  |
| mRE2 | + | + |  |  |  |  | +/- |  | + |
| mRE3 | + |  |  |  |  |  |  |  | + |
| mRE4 | + |  |  | + | + |  |  |  | +/- |
| mRE5 | + |  |  | + |  |  |  |  |  |
|  |  |  |  |  |  |  |  |  |  |
| mRE6 | + | + |  | + |  |  | +/- | + | + |
| mRE7 | + |  |  |  |  |  |  |  |  |
| mRE8 | + | + |  | + | + |  | + |  |  |
| mRE9 |  | + |  |  |  |  | +/- |  |  |
| mRE10 | + |  |  |  |  |  |  |  |  |
| mRE11 | + | + |  | + |  |  | + | + |  |

Murine RE candidates selected based on accessible chromatin in AV junction cardiomyocytes (ATAC_AVJ), EMERGE prediction, or mSTARR_SG4/Wnt activity. Overlap is depicted of candidate regions with ChIP-seq peaks from publicly available datasets for Gata4 (differentiated cardiomyocytes) (Luna-Zurita et al., 2016), Hand2 (E10.5 heart) (Laurent et al., 2017) and H3K27ac (E11.5 heart) (Nord et al., 2013).

**Supplementary Table 4.** Human RE candidates within VR1 and VR2.

|  |  | ***m*RE** |  | **hSTARR** | |  |  |  |  |
| --- | --- | --- | --- | --- | --- | --- | --- | --- | --- |
|  | **RE** | **Liftover** | **EMERGE** | **SG4** | **Wnt** | **SNP** | **trait** | **p-value** |  |
|  | *h*RE3 | *m*RE2 | + |  |  | rs12314403 | PR | 1.7x10^-31^ |  |
|  | *h*RE4 |  |  |  | + |  |  |  |  |
|  | *h*RE5 | *m*RE3 |  |  | + | rs7310299 | PR | 4.7x10^-2^ |  |
|  | *h*RE6 | *m*RE4 |  |  |  | rs10744836 | PR;QRS | 5.0x10^-27^;2.3x10^-11^ |  |
|  | *h*RE7 | *m*RE5 |  |  |  | rs1896329 | PR;QRS | 7.4x10^-28^;3.1x10^-10^ |  |
|  |  |  |  |  |  | rs2114669 | PR | 2.9x10^-2^ |  |
|  |  |  |  |  |  | rs2384552 | PR;QRS | 2.1x10^-23^;4.4x10^-9^ |  |
|  | *h*RE8 |  |  | + |  | rs6489991 | PR | 7.5x10^-6^ |  |
|  |  |  |  |  |  | rs1991391 | PR | 4.4x10^-7^ |  |
|  |  |  |  |  |  | rs2384550 | PR | 4.1x10^-7^ |  |
|  |  |  |  |  |  | rs6489992 | PR | 6.7x10^-6^ |  |
|  | *h*RE9 |  |  |  | + | rs1896312 | PR;QRS | 1.2x10^-34^;5.0x10^-11^ |  |
|  |  |  |  |  |  | rs1863707 | PR | 1.4x10^-11^ |  |
|  |  |  |  |  |  |  |  |  |  |
|  |  |  |  |  |  |  |  |  |  |
|  | *h*RE10 | *m*RE6 |  |  |  |  |  |  |  |
|  | *h*RE11 | *m*RE7 |  |  |  | rs2062717 | PR | 5.3x10^-11^ |  |
|  | *h*RE12 |  |  | + |  |  |  |  |  |
|  | *h*RE13 |  |  |  | + | rs7314370 | PR | 1.5x10^-3^ |  |
|  | *h*RE14 | *m*RE8 | + |  |  | rs11067264 | PR | r^2^ 0.82 w/ rs11067251; rs6488974 |  |
|  | *h*RE15 | *m*RE9 |  |  |  | rs11067258 | PR | 4.4x10^-3^ |  |
|  |  |  |  |  |  | rs2062712 | PR | 4.2x10^-2^ |  |
|  | *h*RE16 |  |  |  | + | rs11067251 | PR | 3.2x10^-17^ |  |
|  |  |  |  |  |  | rs12322585 | PR | 9.2x10^-4^ |  |
|  |  |  |  |  |  | rs12297161 | PR | 7.0x10^-4^ |  |
|  |  |  |  |  |  | rs2384479 | PR | 3.3x10^-16^ |  |
|  |  |  |  |  |  | rs7316851 | PR | 1.4x10^-3^ |  |
|  |  |  |  |  |  | rs6489974 | PR | 4.5x10^-17^ |  |
|  |  |  |  |  |  | rs16944593 | PR | 6.9x10^-2^ |  |
|  |  |  |  |  |  | rs6489975 | PR | 7.2x10^-2^ |  |
|  | *h*RE17 | *m*RE10 |  |  |  |  |  |  |  |
|  | *h*RE18 | *m*RE11 | + | + | + | rs35789998 |  | r^2^ 0.52 w/ rs11067251 |  |
|  |  |  |  |  |  | rs1354155 |  | r^2^ 0.53 w/ rs11067251 |  |
|  |  |  |  |  |  | rs61931165 |  | r^2^ 0.53 w/ rs11067251 |  |
|  | *h*RE19 |  |  |  | + |  |  |  |  |
|  | *h*RE20 |  |  | + |  |  |  |  |  |
|  | *h*RE21 |  |  | + |  |  |  |  |  |
|  | *h*RE22 |  |  | + |  |  |  |  |  |
|  |  |  |  |  |  |  | |  |  |

Human RE candidates selected based on mouse RE homology (mm9 liftover), EMERGE prediction, or hSTARR_SG4/Wnt activity. Regions overlapping GWAS SNPs (SNP id, trait association and respective p-value are listed) were selected for further analysis.

**Supplementary Table 5.** TALEN/CRISPR target sequences for deletion of VR1 and VR2 from the murine genome.

| **Target site** | **Target sequence** |  |
| --- | --- | --- |
| TALEN_VR1_1 | TTCTCAGGGGCCTTTCAAagaagaaagcttcgcAGCCCAGCTTCATCCAGAA | |
| TAL1 RVD sequence | NG-HD-NG-HD-NI-NH-NH-NH-NH-HD-HD-NG-NG-NG-HD-NI-NI | |
| TAL2 RVD sequence | NG-HD-NG-NH-NH-NI-NG-NH-NI-NI-NH-HD-NG-NH-NH-NH-HD-NG | |
|  |  | |
| TALEN_VR1_2 | TGTGGGAGAACCCTGCCccctctgcagaagcaGCTGGAGCCTTCTGCATGGA | |
| TALEN_VR1_2_RVD sequence TAL1 | NH-NG-NH-NH-NH-NI-NH-NI-NI-HD-HD-HD-NG-NH-HD-HD | |
| TALEN_VR1_2_RVD sequence TAL2 | HD-HD-NI-NG-NH-HD-NI-NH-NI-NI-NH-NH-HD-NG-HD-HD-NI-NH-HD | |
|  |  |  |
|  |  |  |
| **Target site** | **Target sequence** |  |
| CRISPR_VR2_1 | GGCTTTGAAGAAGAGTATCA |  |
| sgRNA oligonucleotide sequence 1 | TAGGCTTTGAAGAAGAGTATCA |  |
| sgRNA oligonucleotide sequence 2 | AAACTGATACTCTTCTTCAAAG |  |
|  |  |  |
| CRISPR_VR2_2 | GGGTTTCTCCTGGAGGGTCG |  |
| sgRNA oligonucleotide sequence 1 | TAGGGTTTCTCCTGGAGGGTCG |  |
| sgRNA oligonucleotide sequence 2 | AAACCGACCCTCCAGGAGAAAC |  |

**Supplementary Table 6.** qPCR primer sequences.

| **Primer** | **Primer sequence (5'--3')** |
| --- | --- |
| Tbx3_F | GTCTCAGGCCTAGAATCCAC |
| Tbx3_R | GCCATGTATGTGTAGGGGTA |
| Tbx2_F | CGCAGGCGGGCTAGGTC |
| Tbx2_R | AAGAGGCCTCCGAAAGTGGG |
| Tbx5_F | CCCGGAGACAGCTTTTATCG |
| Tbx5_F | TGGTTGGAGGTGACTTTGTG |
| Med13l_F | CACGGAGTTTAGGATGGAAGTGG |
| Med13l_R | AAGGCTGGAACTGCGGCACTTT |
| Rbm19_F | TACGGAACCTGTCCTACACCAG |
| Rbm19_R | AGGTGACGAAGGCAAAGCCCTT |
| Isl1_F | GCAACCCAACGACAAAACTAA |
| Isl1_R | CCATCATGTCTCTCCGGACT |
| Eef2_F | TGGAGCCTATCTATCTGGTGG |
| Eef2_R | GTCTCAGCTACCACTTGGCT |
| Scn5a_F | GGGACTCATTGCCTACATGA |
| Scn5a_R | GCACTGGGAGGTTATCACTG |
| Cacna1g_F | GAAGGTGTCAATAGCCCTGA |
| Cacna1g_R | GACACCAGACTTCCTCACAG |
| Hcn4_F | GCGTGCTCACTAAGGGCAAC |
| Hcn4_R | GGCAATAAGTATCCGCTCTGAC |
| Gja5_F | GCCTGAAGAAGCCAACTCCAGG |
| Gja5_R | GCTTGTGGACCTCCTCCAGG |
| Gja1_F | AGTACCCAACAGCAGCAGAC |
| Gja1_R | AAAATGAAGAGCACCGACAG |
| Ryr2_F | CAAATCCTTCTGCTGCCAAG |
| Ryr2_R | CGAGGATGAGATCCAGTTCC |

**Supplementary Table 7.** Overview of BACs used for the generation of STARR-seq libraries

| **Murine BACs** |  |  |
| --- | --- | --- |
| **BAC name** | **Size [bp]** | **Genomic coordinates [mm9]** |
| RP23-27G24 | 237655 | chr5:120519399-120745441 |
| RP23-183L13 | 212708 | chr5:120318246-120519341 |
| RP23-376N10 | 152322 | chr5:120182858-120323567 |
| RP23-366H17 | 171534 | chr5:120038644-120198565 |
| RP23-459M16 | 181931 | chr5:119874765-120045083 |
| RP24-250N7 | 151066 | chr5:119741090-119878692 |
| RP23-308O19 | 206616 | chr5:119565148-119760151 |
| RP24-371D5 | 208802 | chr5:119387623-119582961 |
| RP23-296H19 | 209394 | chr5:119203312-119401093 |
| RP23-34B9 | 207424 | chr5:119035452-119231263 |
| RP23-335A14 | 227475 | chr5:118828656-119044518 |
|  |  |  |
| **Human BACs** |  |  |
| **BAC name** | **Size [bp]** | **Genomic coordinates [hg19]** |
| RP11-597C16 | 168854 | chr12:116697763-116866617 |
| RP11-379F8 | 167988 | chr12:116533799-116701787 |
| RP11-1130I19 | 141901 | chr12:116391900-116533801 |
| RP11-412G23 | 169878 | chr12:116224958-116394836 |
| RP11-36E12 | 149382 | chr12:116075502-116224884 |
| RP11-346N22 | 162603 | chr12:115966759-116129362 |
| RP11-809J20 | 180445 | chr12:115786239-115966684 |
| RP11-42O13 | 155240 | chr12:115640724-115795964 |
| RP11-992D2 | 185118 | chr12:115458557-115643675 |
| RP11-693B4 | 203971 | chr12:115272432-115476403 |
| RP11-879D18 | 173123 | chr12:115102051-115275174 |
| RP11-125E3 | 165503 | chr12:114956460-115121963 |
| RP11-91M21 | 171499 | chr12:114821528-114993027 |
| RP11-333I15 | 170736 | chr12:114694877-114865613 |
| RP11-100F15 | 185941 | chr12:114546237-114732178 |
| RP11-269C10 | 200600 | chr12:114351967-114552567 |
| RP11-37N9 | 177697 | chr12:114166377-114344074 |

**Supplementary Table 8.** Primer sequences for the amplification of active STARR-seq regions for validation by luciferase reporter assay.

| **Primer name** | **Primer sequence (5'--3')** |
| --- | --- |
| mSTARR_validation_1_F | ACAACAATCTTAGTGCCAGCAA |
| mSTARR_validation_1_R | GCGTCAATCATGGCTTCTCAAC |
| mSTARR_validation_2_F | GCTCACCACAGCCTGTTCA |
| mSTARR_validation_2_R | ATCCTAGCTCAGACTGTCTTCA |
| mSTARR_validation_3_F | CCCTGAGTGTTAGGAGAGTTGT |
| mSTARR_validation_3_R | AATTCACCATGGAGTCAGGTTT |
| mSTARR_validation_4_F | GGGAGATCAGAGGACAACCTAC |
| mSTARR_validation_4_R | TTCCGTGACCCAAAGTTCTCTC |
| mSTARR_validation_5_F | CTTTCACTTCCCTGGTCCACTT |
| mSTARR_validation_5_R | TCAGGGCTGGAAGGAGAAGATA |
| mSTARR_validation_6_F | TGGTTCCTGTAGGAGAGGAGAG |
| mSTARR_validation_6_R | TTTGTTTGGCCAGAGAACAACC |
| mSTARR_validation_7_F | TATGTGGGTGCTGGGAATTCAA |
| mSTARR_validation_7_R | AAGGATCCAGTCTCTGGTCTCA |
| mSTARR_validation_8_F | ATCCAGTCCACTGTCTCAGCAA |
| mSTARR_validation_8_R | ACTCTGCCCTTGACAATTCTGT |
| mSTARR_validation_9_F | GGGTTACAGGTGTGTGTCACTA |
| mSTARR_validation_9_R | TGACTACGGAGTTAGTTCAAGG |
|  |  |
| hSTARR_validation_1_F | CACAACAGAAAGGAAGCTCACA |
| hSTARR_validation_1_R | GCAGAGGCATAATGAACAGGTA |
| hSTARR_validation_2_F | GCATCTGTGTTCATCAAGGATAT |
| hSTARR_validation_2_R | CATGACCTTGCTCTTGACTGCT |
| hSTARR_validation_3_F | GCAGCAACCCATCCCAAAAC |
| hSTARR_validation_3_R | GGACCAGCCGTTTCTCTGTTA |
| hSTARR_validation_4_F | GGGGTAGCAGAGTGATGGAA |
| hSTARR_validation_4_R | CCCGCCCTGAATTTGACTTTTC |
| hSTARR_validation_5_F | TGACAACTTCACCTCTCTGCTT |
| hSTARR_validation_5_R | GCCCTCTACCAACTCGCATT |
| hSTARR_validation_6_F | ACCACTCCATCCCTGCTAAC |
| hSTARR_validation_6_R | GCATGGACAAAGGACAGGGAA |
| hSTARR_validation_7_F | AACAGGCAGACCCTCATCAA |
| hSTARR_validation_7_R | GCTCACAAGGTGGATCCCAA |
| hSTARR_validation_8_F | TTCTCTCGGAGAAGCCAGGTT |
| hSTARR_validation_8_R | TTGAGCCAATGCTCCAACTCA |
| hSTARR_validation_9_F | TGTAAGCATGTGCCTAGTTAGTA |
| hSTARR_validation_9_R | AGACTGGGATTGAGGACATGC |
| hSTARR_validation_10_F | GACAAGGCCGATGGATGCTT |
| hSTARR_validation_10_R | GCATCTGACAACCTCAGAACC |

**Supplementary Table 8,** continued

| **Primer name** | **Primer sequence (5’—3’)** |
| --- | --- |
| mSTARR_validation_neg1_F | AATCCTTCCAGCTTCCTTCCTG |
| mSTARR_validation_neg1_R | TATTGGAAAGAACGCCTGTGGA |
| mSTARR_validation_neg2_F | GAGCCCAGATGTTAAACCCTCA |
| mSTARR_validation_neg2_R | GGGTTCAGAAGGTAGATTGGCA |
| mSTARR_validation_neg3_F | GGATGTCAGTGAGGTGGGATTT |
| mSTARR_validation_neg3_R | TTAGAGGGTGGAGACAGGGTAG |
| mSTARR_validation_neg4_F | GAGCTTTCTCCCAACCCACATA |
| mSTARR_validation_neg4_R | AATGTGGACAGGATGAGGTCAC |
| mSTARR_validation_neg5_F | GCTGTCTTCAGACACTCCAGAA |
| mSTARR_validation_neg5_R | ACTGCTGAGTCACACCTACAAG |
| hSTARR_validation_neg1_F | CACCCTCTTGGATGGATTGTGA |
| hSTARR_validation_neg1_R | ACCTTACCCAAGATGTGCCAAT |
| hSTARR_validation_neg2_F | CAGGTGGAAAGGGCTCAGTAAT |
| hSTARR_validation_neg2_R | GGAGTTTGGCTCCATCAGGTAA |
| hSTARR_validation_neg3_F | ATAAGGAAGGGCACAAGACTGG |
| hSTARR_validation_neg3_R | ATGGACAAATGGAAGGCTGGAT |
| hSTARR_validation_neg4_F | TCTTGCTAAATCACTCGGCTGT |
| hSTARR_validation_neg4_R | TGCCTGTTACCTGTCATTCTCC |
| hSTARR_validation_neg5_F | CTGGGCTGACTCTGAGACATTT |
| hSTARR_validation_neg5_R | AGGGTAGCATGCCAATTCTTCA |

**Supplementary Table 9.** Primer sequences for the amplification of RE candidate fragments.

| **Primer name** | **Primer sequence (5'--3')** |
| --- | --- |
| *h*RE3_F | AATGGGCTCCTGTCAATATCC |
| *h*RE3_R | GAAGGACCAAGCCTGGATGT |
| *h*RE5_F | AAGCTGCAAGTCAAAAGGCATC |
| *h*RE5_R | CTGGCATTGCTACCTAACATC |
| *h*RE6_F | TACGGGACAGACCTGACATC |
| *h*RE6_R | AAATCCCAGAGGCCAAAGGC |
| *h*RE7_F | GAGTTGGAGGCAACTTTGAAG |
| *h*RE7_R | AATCACAGAGGGCAGAGGTC |
| *h*RE8_F | CTCCACTTAGACTCAAATCCTG |
| *h*RE8_R | GTTGGCTCACATTGTAGAGTG |
| *h*RE9_F | CTTCTTCCAATGCCCTTTTGC |
| *h*RE9_R | CAACTGTTTTGTGGCATTGAGC |
| *h*RE11_F | CCTTCCCAAGACTTCTAATGTG |
| *h*RE11_R | GGAGAAGGGGAAAGAGAGTG |
| *h*RE13_F | GCTGAAGATGGAGTGCAATAG |
| *h*RE13_R | CCAACATCTCAGGCCTTCTC |
| *h*RE15_F | GTGCCTGACATCAAATACTTGG |
| *h*RE15_R | TGGACATGGCTGAAGAAACCA |
| *h*RE16_F | CCACTCAACTGTAATGGCTTC |
| *h*RE16_R | TATGTTACCCAGGCATAGTGAG |
| *h*RE19_F | GAGATGGAGAAGACCTGGAC |
| *h*RE19_R | CGGCTTGGATCTGCATAGAC |
| *h*RE14_F | CCAGGGTACCCAGCTTATAG |
| *h*RE14_R | CCTGGCTAGATGTTAAAAGCTA |
| *h*RE18_F | GGCAGATTGTCCGAGGTTAG |
| *h*RE18_R | AGAGAGGTTCTCATTCATTAGG |

**Supplementary Table 10.** Mouse functional genomic datasets included in merge as possible predictors.

| **Dataset** | **Type** | **Genome** | **Tissue** | **Overall** | **Stage** | **Source** | **Deposit** | **# of Peaks** |
| --- | --- | --- | --- | --- | --- | --- | --- | --- |
| Conservation | PhastCons | mm9 | all | all | all | Siepel lab | UCSC | 972010 |
| DHSs | Dnase1 hypersensitivity | mm9 | whole_brain | brain | E18.5 | ENCODE | GSE37074 | 143557 |
| H3K27ac | ChIP‐seq | mm9 | whole_brain | brain | E14.5 | ENCODE | GSE49847 | 34012 |
| H3K4me1 | ChIP‐seq | mm9 | whole_brain | brain | E14.5 | ENCODE | GSE49847 | 152915 |
| H3K4me3 | ChIP‐seq | mm9 | whole_brain | brain | E14.5 | ENCODE | GSE49847 | 36227 |
| Pol2 | ChIP‐seq | mm9 | whole_brain | brain | E14.5 | ENCODE | GSE49847 | 17146 |
| Smarca4 | ChIP‐seq | mm9 | forebraim | brain | E11.5 | Pennacchio lab | GSE37151 | 7349 |
| ATAC | ATAC‐seq | mm9 | Ventricle | heart | E12.5 | Christoffels lab | in house | 84667 |
| ATAC | ATAC‐seq | mm9 | right arium | heart | E12.5 | Christoffels lab | in house | 129996 |
| ATAC | ATAC‐seq | mm9 | left atrium | heart | E12.5 | Christoffels lab | in house | 125461 |
| ATAC | ATAC‐seq | mm9 | HL‐1 cells | heart | no stimulation 20k | Christoffels lab | in house | 110206 |
| ATAC | ATAC‐seq | mm9 | HL‐1 cells | heart | no stimulation 50k | Christoffels lab | in house | 150324 |
| ATAC | ATAC‐seq | mm9 | HL‐1 cells | heart | Bmp2 stim 20k | Christoffels lab | in house | 150279 |
| ATAC | ATAC‐seq | mm9 | HL‐1 cells | heart | Bmp2 stim 50k | Christoffels lab | in house | 130868 |
| ATAC | ATAC‐seq | mm9 | atria | heart | Embryonic | Moskowitz | unpublished | 18213 |
| ATAC | ATAC‐seq | mm9 | pSHF | heart | Embryonic | Moskowitz | unpublished | 21318 |
| ATAC | ATAC‐seq | mm9 | ventricles | heart | Embryonic | Moskowitz | unpublished | 33418 |
| Coup‐TFII | ChIP‐seq | mm9 | atria | heart | E14.5 | Tsai lab | GSE46497 | 4359 |
| DHSs | Dnase1 hypersensitivity | mm9 | heart | heart | adult | ENCODE | GSE37074 | 150000 |
| Gata4 | ChIP‐seq | mm9 | heart | heart | adult | Christoffels lab | GSE35151 | 150493 |
| Gata4 | ChIP‐seq | mm9 | heart | heart | E12.5 | Pu lab | GSE52123 | 150033 |
| Gata4 | ChIP‐seq | mm9 | heart | heart | adult | Pu lab | GSE52123 | 150054 |
| Gata4 | ChIP‐exo | mm9 | myocyte cell line | heart | differentiated | Bruneau lab | GSE77548 | 25710 |
| Gata4 | ChIP‐exo | mm9 | myocyte cell line | heart | cardiac_precursor | Bruneau lab | GSE77548 | 52999 |
| H3ac | ChIP‐seq | mm9 | left ventric~~ | heart | adult | Baker lab | GSE63590 | 150201 |
| H3K27ac | ChIP‐seq | mm9 | avc | heart | E10.5 | Christoffels lab | GSE55611 | 87572 |
| H3K27ac | ChIP‐seq | mm9 | right atrium | heart | E10.5 | Christoffels lab | GSE55611 | 67092 |
| H3K27ac | ChIP‐seq | mm9 | heart | heart | adult | ENCODE | GSE49847 | 53034 |
| H3K27ac | ChIP‐seq | mm9 | heart | heart | E14.5 | ENCODE | GSE49847 | 84442 |
| H3K27ac | ChIP‐seq | mm9 | heart | heart | adult | Pennacchio lab | GSE52386 | 150515 |
| H3K27ac | ChIP‐seq | mm9 | heart | heart | E11.5 | Pennacchio lab | GSE52386 | 32289 |
| H3K27ac | ChIP‐seq | mm9 | heart | heart | E14.5 | Pennacchio lab | GSE52386 | 31815 |
| H3K27ac | ChIP‐seq | mm9 | heart | heart | E17.5 | Pennacchio lab | GSE52386 | 23210 |
| H3K27ac | ChIP‐seq | mm9 | heart | heart | P0 | Pennacchio lab | GSE52386 | 39133 |
| H3K27ac | ChIP‐seq | mm9 | heart | heart | P7 | Pennacchio lab | GSE52386 | 36447 |
| H3K27ac | ChIP‐seq | mm9 | heart | heart | P21 | Pennacchio lab | GSE52386 | 7376 |
| H3K27me3 | ChIP‐seq | mm9 | heart | heart | adult | ENCODE | GSE49847 | 21876 |
| H3K4me1 | ChIP‐seq | mm9 | heart | heart | adult | ENCODE | GSE49847 | 150914 |
| H3K4me3 | ChIP‐seq | mm9 | heart | heart | adult | ENCODE | GSE49847 | 53034 |
| Hand2 (rep1) | ChIP‐seq | mm9 | heart | heart | adult | Zeller lab | confidential? | 150002 |
| Hand2 (rep2) | ChIP‐seq | mm9 | heart | heart | adult | Zeller lab | confidential? | 20446 |
| Hif1a rep1 | ChIP‐seq | mm9 | heart | heart | E12.5 | Keck institute | GSE61247 | 17289 |
| Hopx | ChIP‐seq | mm9 | heart | heart | E9.5 | Won lab | GSE67251 | 3774 |
| Hopx with EGS | ChIP‐seq | mm9 | heart | heart | E9.5 | Won lab | GSE67251 | 178 |
| Isl1 | ChIP‐seq | mm9 | sinus node | heart | p3 | Evans lab | GSE68974 | 2322 |
| Mef2a | ChIP‐exo | mm9 | C2C12 cell line | heart | myoblast | McDermott lab | GSE61204 | 2796 |
| Nkx2‐5 | ChIP‐seq | mm9 | heart | heart | adult | Christoffels lab | GSE35151 | 150227 |
| Nkx2‐5 | ChIP‐seq | mm9 | heart | heart | E12.5 | Chen lab | GSE70332 | 3967 |
| Nkx2‐5 | ChIP‐exo | mm9 | myocyte cell line | heart | differentiated | Bruneau lab | GSE77548 | 62218 |
| Nkx2‐5 | ChIP‐exo | mm9 | myocyte cell line | heart | cardiac_precursor | Bruneau lab | GSE77548 | 9463 |
| p300 (rep1) | ChIP‐seq | mm9 | heart | heart | adult | ENCODE | GSE49847 | 137325 |
| p300 (rep2) | ChIP‐seq | mm9 | heart | heart | adult | ENCODE | GSE49847 | 116158 |
| Pol2 | ChIP‐seq | mm9 | heart | heart | adult | ENCODE | GSE49847 | 58637 |
| RBFox2 | ChIP‐seq | mm9 | heart | heart | 9w | Palade lab | GSE57926 | 151144 |
| Shox2 | ChIP‐seq | mm9 | heart | heart | E12.5 | Chen lab | GSE70332 | x |
| Smarca4 | ChIP‐seq | mm9 | heart | heart | E11.5 | Pennacchio lab | GSE37151 | 13775 |
| SOX9 | ChIP‐seq | mm9 | AVC~~ | heart | E12.5 | Fox lab | GSE73225 | x |
| ß‐catenin biotin | ChIP‐seq | mm9 | mESC | heart | blastocyst | McMahon lab | GSE43565 | 20408 |
| ß‐catenin flag | ChIP‐seq | mm9 | mESC | heart | blastocyst | McMahon lab | GSE43565 | 20294 |
| Suz12 | ChIP‐seq | mm9 | heart | heart | 9w | Palade lab | GSE57926 | 22476 |
| Tbx20 | ChIP‐seq | mm9 | heart | heart | adult | Evans lab | GSE30943 | 4011 |
| TBX3 | ChIP‐seq | mm9 | heart | heart | adult | Christoffels lab | GSE44821 | 150859 |
| Tbx5 | ChIP‐seq | mm9 | HL‐1 cells | heart | cell line | Pu lab | GSE21529 | 48221 |
| Tbx5 | ChIP‐exo | mm9 | myocyte cell line~ | heart | differentiated | Bruneau lab | GSE77548 | 45540 |
| Tbx5 | ChIP‐exo | mm9 | myocyte cell line~ | heart | cardiac_precursor | Bruneau lab | GSE77548 | 8587 |
| Tcf3 | ChIP‐seq | mm9 | mESC | heart | blastocyst | Whitehead Inst. | GSE11724 | 7336 |
| Smarca4 | ChIP‐seq | mm9 | limb | limb | E11.5 | Pennacchio lab | GSE37151 | 5251 |
| SOX9 | ChIP‐seq | mm9 | Limb | limb | E12.5 | Fox lab | GSE73225 | 151213 |
| ATAC | ATAC‐seq | mm9 | liver | liver | E12.5 | Christoffels lab | in house | 70760 |
| Hey2 | ChIP‐seq | mm9 | mESC | stem cell | blastocyst | Gessler | TBA | 99750 |
| H3K27ac | ChIP‐seq | mm9 | forebrain | brain | adult | Pennacchio lab | GSE52386 | 33178 |
| H3K27ac | ChIP‐seq | mm9 | forebrain | brain | E11.5 | Pennacchio lab | GSE52386 | 15823 |
| H3K27ac | ChIP‐seq | mm9 | forebrain | brain | E14.5 | Pennacchio lab | GSE52386 | 27288 |
| H3K27ac | ChIP‐seq | mm9 | forebrain | brain | E17.5 | Pennacchio lab | GSE52386 | 20450 |
| H3K27ac | ChIP‐seq | mm9 | forebrain | brain | P0 | Pennacchio lab | GSE52386 | 30308 |
| H3K27ac | ChIP‐seq | mm9 | forebrain | brain | P7 | Pennacchio lab | GSE52386 | 36771 |
| H3K27ac | ChIP‐seq | mm9 | forebrain | brain | P21 | Pennacchio lab | GSE52386 | 15246 |
| H3K27ac | ChIP‐seq | mm9 | forelimb | limb | E11.5 | Menke lab | GSE64055 | 40303 |
| H3K27ac | ChIP‐seq | mm9 | hindlimb | limb | E11.5 | Menke lab | GSE64055 | 35166 |
| Pol2 | ChIP‐seq | mm9 | limb | limb | E14.5 | ENCODE | GSE49847 | 14426 |
| H3K4me1 | ChIP‐seq | mm9 | limb | limb | E14.5 | ENCODE | GSE49847 | 150360 |
| H3K4me3 | ChIP‐seq | mm9 | limb | limb | E14.5 | ENCODE | GSE49847 | 19259 |
| H3K27ac | ChIP‐seq | mm9 | limb | limb | E14.5 | ENCODE | GSE49848 | 30543 |
| DHSs | Dnase1 hypersensitivity | mm9 | forelimb | limb | E11.5 | ENCODE | GSE37074 | 151669 |
| DHSs | Dnase1 hypersensitivity | mm9 | hindlimb | limb | E11.5 | ENCODE | GSE37074 | 150814 |
| H3K27ac | ChIP‐seq | mm9 | forebrain | brain | E12.5 | Rockowitz | GSE66961 | 26863 |
| H3K4me1 | ChIP‐seq | mm9 | forebrain | brain | E12.5 | Rockowitz | GSE66961 | 40773 |
| H3K4me3 | ChIP‐seq | mm9 | forebrain | brain | E12.5 | Rockowitz | GSE66961 | 15407 |

**Supplementary Table 10,** continued.

| **Dataset** | **Type** | **Genome** | **Tissue** | **Overall** | **Stage** | **Source** | **Deposit** | **# of peaks** |
| --- | --- | --- | --- | --- | --- | --- | --- | --- |
| H3K27me3 | ChIP‐seq | mm9 | forebrain | brain | E12.5 | Rockowitz | GSE66961 | 25473 |
| Pol2 | ChIP‐seq | mm9 | forebrain | brain | E12.5 | Rockowitz | GSE66961 | 1704 |
| H3K4me1 | ChIP‐seq | mm9 | midbrain | brain | E11.5 | Endo | GSE49652 | 19384 |
| H3K27ac | ChIP‐seq | mm9 | midbrain | brain | E11.5 | Endo | GSE49652 | 22752 |
| p300 | ChIP‐seq | mm9 | midbrain | brain | E11.5 | Pennacchio lab | GSE22549 | 2786 |
| p300 | ChIP‐seq | mm9 | heart | heart | E11.5 | Pennacchio lab | GSE22549 | 3597 |
| p300 | ChIP‐seq | mm9 | forebrain | brain | E11.5 | Pennacchio lab | GSE13845 | 2454 |
| p300 | ChIP‐seq | mm9 | midbrain | brain | E11.5 | Pennacchio lab | GSE13845 | 562 |
| p300 | ChIP‐seq | mm9 | limb | limb | E11.5 | Pennacchio lab | GSE13845 | 2106 |
| DHSs | Dnase1 hypersensitivity | mm9 | cerebellum | brain | P7 | Crawford | GSE60731 | 100000 |
| DHSs | Dnase1 hypersensitivity | mm9 | cerebellum | brain | P14 | Crawford | GSE60731 | 100000 |
| DHSs | Dnase1 hypersensitivity | mm9 | cerebellum | brain | Adult | Crawford | GSE60731 | 100000 |

**Supplementary Table 11.** Human functional genomic datasets included in merge as possible predictors.

| **Dataset** | **Type** | **Genome** | **Tissue** | **Stage** | **Source** | **Deposit** | **# of Peaks** |
| --- | --- | --- | --- | --- | --- | --- | --- |
| Conservation | PhastCons | hg19 | all | all | Siepel lab | UCSC | 10121233 (174584)* |
| CTCF | ChIP‐seq | hg19 | HCM cells | cell line | ENCODE | GSE30263 | 41966 |
| DHSs | Dnase1 hypersensitivity | hg19 | cerebellum | adult | ENCODE | GSE32970 | 104575 |
| DHSs | Dnase1 hypersensitivity | hg19 | cerebrum | adult | ENCODE | GSE32970 | 135930 |
| DHSs | Dnase1 hypersensitivity | hg19 | frontal_cortex | adult | ENCODE | GSE32970 | 110989 |
| DHSs | Dnase1 hypersensitivity | hg19 | HCM cells | cell line | ENCODE | GSE26328 | 317895 |
| DHSs | Dnase1 hypersensitivity | hg19 | heart | adult | ENCODE | GSE32970 | 124949 |
| H3K27ac | ChIP‐seq | hg19 | brain | fetal 12w post‐gest. | Guo lab | GSE63634 | 77355 |
| H3K27ac | ChIP‐seq | hg19 | cerebellum | adult | Creyghton lab | GSE40465 | 174090 |
| H3K27ac | ChIP‐seq | hg19 | fibroblast cell line | induced myocytes D6 | Cao lab | GSE78096 | 33954 |
| H3K27ac | ChIP‐seq | hg19 | fibroblast cell line | induced myocytes D11 | Cao lab | GSE78096 | 36649 |
| H3K27ac | ChIP‐seq | hg19 | heart | fetal 12w post‐gest. | Guo lab | GSE63634 | 49185 |
| H3K27ac | ChIP‐seq | hg19 | iPSC | induced myocytes | Sakabe lab | GSE77267 | 19528 |
| H3K27ac | ChIP‐seq | hg19 | left ventricle | adult | Ren lab | GSE16256 | 59070 |
| H3K27ac | ChIP‐seq | hg19 | limbs | gest. E33 | Cotney lab | GSE42413 | 72841 |
| H3K27ac | ChIP‐seq | hg19 | limbs | gest. E41 | Cotney lab | GSE42413 | 42284 |
| H3K27ac | ChIP‐seq | hg19 | limbs | gest. E44 | Cotney lab | GSE42413 | 69409 |
| H3K27ac | ChIP‐seq | hg19 | limbs | gest. E47 | Cotney lab | GSE42413 | 54981 |
| H3K27ac | ChIP‐seq | hg19 | liver | fetal 12w post‐gest. | Guo lab | GSE63634 | 27741 |
| H3K27ac | ChIP‐seq | hg19 | right ventricle | adult | Ren lab | GSE16256 | 126802 |
| H3K27me3 | ChIP‐seq | hg19 | brain | fetal 12w post‐gest. | Guo lab | GSE63634 | 50180 |
| H3K27me3 | ChIP‐seq | hg19 | fibroblast cell line | induced myocytes D6 | Cao lab | GSE78096 | 275 |
| H3K27me3 | ChIP‐seq | hg19 | fibroblast cell line | induced myocytes D11 | Cao lab | GSE78096 | 173 |
| H3K27me3 | ChIP‐seq | hg19 | heart | fetal 12w post‐gest. | Guo lab | GSE63634 | 34795 |
| H3K27me3 | ChIP‐seq | hg19 | hESC | induced myocytes 14d | Murry lab | SRR577565 | 86945 |
| H3K27me3 | ChIP‐seq | hg19 | iPSC | induced myocytes | Sakabe lab | GSE77267 | 8952 |
| H3K27me3 | ChIP‐seq | hg19 | liver | fetal 12w post‐gest. | Guo lab | GSE63634 | 41712 |
| H3K36me3 | ChIP‐seq | hg19 | hESC | induced myocytes 14d | Murry lab | SRR577574 | 118126 |
| H3K4me1 | ChIP‐seq | hg19 | brain | fetal 12w post‐gest. | Guo lab | GSE63634 | 58426 |
| H3K4me1 | ChIP‐seq | hg19 | heart | fetal (d105) | BROAD institute | GSE17312 | 113536 |
| H3K4me1 | ChIP‐seq | hg19 | heart | fetal 12w post‐gest. | Guo lab | GSE63634 | 59026 |
| H3K4me1 | ChIP‐seq | hg19 | iPSC | induced myocytes | Sakabe lab | GSE77267 | 13902 |
| H3K4me1 | ChIP‐seq | hg19 | liver | fetal 12w post‐gest. | Guo lab | GSE63634 | 43426 |
| H3K4me3 | ChIP‐seq | hg19 | brain | fetal 12w post‐gest. | Guo lab | GSE63634 | 16745 |
| H3K4me3 | ChIP‐seq | hg19 | fibroblast cell line | induced myocytes D6 | Cao lab | GSE78096 | 18720 |
| H3K4me3 | ChIP‐seq | hg19 | fibroblast cell line | induced myocytes D11 | Cao lab | GSE78096 | 19871 |
| H3K4me3 | ChIP‐seq | hg19 | HCM cells | cell line | ENCODE | GSE35583 | 49247 |
| H3K4me3 | ChIP‐seq | hg19 | heart | fetal (d105) | BROAD institute | GSE17312 | 1238 |
| H3K4me3 | ChIP‐seq | hg19 | heart | fetal 12w post‐gest. | Guo lab | GSE63634 | 86937 |
| H3K4me3 | ChIP‐seq | hg19 | hESC | induced myocytes 14d | Murry lab | SRR577568 | 26960 |
| H3K4me3 | ChIP‐seq | hg19 | iPSC | induced myocytes | Sakabe lab | GSE77267 | 10760 |
| H3K4me3 | ChIP‐seq | hg19 | liver | fetal 12w post‐gest. | Guo lab | GSE63634 | 11799 |
| H3K9ac | ChIP‐seq | hg19 | heart | fetal (d105) | BROAD institute | GSE17312 | 49876 |
| p300 | ChIP‐seq | hg19 | cortex | fetal (gest. week 20) | Pennacchio lab | GSE42881 | 642876 |
| p300 | ChIP‐seq | hg19 | heart | adult | Pennacchio lab | GSE32587 | 139824 |
| p300 | ChIP‐seq | hg19 | heart | fetal (gest. week 16) | Pennacchio lab | GSE32587 | 108384 |
| Pol2 | ChIP‐seq | hg19 | heart | fetal (gest. week 16) | Pennacchio lab | GSE32587 | 223086 |
| Pol2 | ChIP‐seq | hg19 | iPSC | induced myocytes | Sakabe lab | GSE77267 | 11332 |
| ATAC | ATAC‐seq | hg19 | SANLPCs | induced SAN myocytes | Protze lab | unpublished | 45389 |
| ATAC | ATAC‐seq | hg19 | hESC | 0d H1 differentiated myocytes | Snyder lab | GSE85330 | 54378 |
| ATAC | ATAC‐seq | hg19 | hESC | 2d H1 differentiated myocytes | Snyder lab | GSE85330 | 21979 |
| ATAC | ATAC‐seq | hg19 | hESC | 4d H1 differentiated myocytes | Snyder lab | GSE85330 | 28991 |
| ATAC | ATAC‐seq | hg19 | hESC | 30d H1 differentiated myocytes | Snyder lab | GSE85330 | 11931 |
| ATAC | ATAC‐seq | hg19 | hESC | 0d H9 differentiated myocytes | Snyder lab | GSE85330 | 28417 |
| ATAC | ATAC‐seq | hg19 | hESC | 2d H9 differentiated myocytes | Snyder lab | GSE85330 | 10980 |
| ATAC | ATAC‐seq | hg19 | hESC | 4d H9 differentiated myocytes | Snyder lab | GSE85330 | 26580 |
| ATAC | ATAC‐seq | hg19 | hESC | 30d H9 differentiated myocytes | Snyder lab | GSE85330 | 24661 |
| ATAC | ATAC‐seq | hg19 | iPSC | 0d C15 induced myocytes | Snyder lab | GSE85330 | 37100 |
| ATAC | ATAC‐seq | hg19 | iPSC | 2d C15 induced myocytes | Snyder lab | GSE85330 | 35192 |
| ATAC | ATAC‐seq | hg19 | iPSC | 4d C15 induced myocytes | Snyder lab | GSE85330 | 13920 |
| ATAC | ATAC‐seq | hg19 | iPSC | 30d C15 induced myocytes | Snyder lab | GSE85330 | 16191 |
| ATAC | ATAC‐seq | hg19 | iPSC | 0d C20 induced myocytes | Snyder lab | GSE85330 | 27217 |
| ATAC | ATAC‐seq | hg19 | iPSC | 2d C20 induced myocytes | Snyder lab | GSE85330 | 22065 |
| ATAC | ATAC‐seq | hg19 | iPSC | 4d C20 induced myocytes | Snyder lab | GSE85330 | 51994 |
| ATAC | ATAC‐seq | hg19 | iPSC | 30d C20 induced myocytes | Snyder lab | GSE85330 | 19602 |
| ATAC | ATAC‐seq | hg19 | hESC | cardiac mesoderm H7 | Weissman lab | GSE85066 | 180380 |
| ATAC | ATAC‐seq | hg19 | left ventricle | induced LV myocytes | Protze lab | unpublished | 38335 |
| ATAC | ATAC‐seq | hg19 | left atrium | adult | Martin lab | unpublished | 55045 |
| DHSs | Dnase1 hypersensitivity | hg19 | limbs | fetal | ENCODE | GSE90405 | 95652 |
| Med1 | ChIP‐seq | hg19 | iPSC | induced myocytes 32d | Srivastava lab | GSE85631 | 15400 |

*number of ultra‐conserved phastCons (score≥600)
