# Supplementary material for: Trait-associated noncoding variant regions affect TBX3 regulation and cardiac conduction
Source: eLife. 2020 Jul 16;9:e56697. doi: 10.7554/eLife.56697 (PMC7365664; doi:10.7554/eLife.56697)
Supplement: Transparent reporting form [file elife-56697-transrepform.docx]

***eLife’s* transparent reporting form**

We encourage authors to provide detailed information *within their submission* to facilitate the interpretation and replication of experiments. Authors can upload supporting documentation to indicate the use of appropriate reporting guidelines for health-related research (see [EQUATOR Network](http://www.equator-network.org/%20)), life science research (see the [BioSharing Information Resource](https://biosharing.org/)), or the [ARRIVE guidelines](http://www.plosbiology.org/article/info:doi/10.1371/journal.pbio.1000412) for reporting work involving animal research. Where applicable, authors should refer to any relevant reporting standards documents in this form.

If you have any questions, please consult our Journal Policies and/or contact us: [editorial@elifesciences.org](mailto:editorial@elifesciences.org).

**Sample-size estimation**

- You should state whether an appropriate sample size was computed when the study was being designed
- You should state the statistical method of sample size computation and any required assumptions
- If no explicit power analysis was used, you should describe how you decided what sample (replicate) size (number) to use

Please outline where this information can be found within the submission (e.g., sections or figure legends), or explain why this information doesn’t apply to your submission:

STARR-seq experiments were performed in duplicates, and aimed to reach >5*10^6 SR50 reads per experiment according to Arnold et al. 2013. CRISPR- and TALEN-mediated genome editing was performed to obtain at least two founder lines per deletion to rule out off-target effects and confirm the effect of the deletion in independent lines. For other experiments, sample size was not pre-determined with any statistical test, but rather estimated based on previous experiments.

**Replicates**

- You should report how often each experiment was performed
- You should include a definition of biological versus technical replication
- The data obtained should be provided and sufficient information should be provided to indicate the number of independent biological and/or technical replicates
- If you encountered any outliers, you should describe how these were handled
- Criteria for exclusion/inclusion of data should be clearly stated
- High-throughput sequence data should be uploaded before submission, with a private link for reviewers provided (these are available from both GEO and ArrayExpress)

Please outline where this information can be found within the submission (e.g., sections or figure legends), or explain why this information doesn’t apply to your submission:

Information on replicates per experiments can be found in the respective figure legends and in the Materials & Methods section. STARR-seq and ATAC-seq data has been deposited in the GEO database, under GEO-accession numbers GSE145257 (available from April 30, 2020) and GSE121464, respectively.

**Statistical reporting**

- Statistical analysis methods should be described and justified
- Raw data should be presented in figures whenever informative to do so (typically when N per group is less than 10)
- For each experiment, you should identify the statistical tests used, exact values of N, definitions of center, methods of multiple test correction, and dispersion and precision measures (e.g., mean, median, SD, SEM, confidence intervals; and, for the major substantive results, a measure of effect size (e.g., Pearson's r, Cohen's d)
- Report exact p-values wherever possible alongside the summary statistics and 95% confidence intervals. These should be reported for all key questions and not only when the p-value is less than 0.05.

Please outline where this information can be found within the submission (e.g., sections or figure legends), or explain why this information doesn’t apply to your submission:

Information regarding the statistical analysis methods used for each experiment can be found in the figure legends, and in the Results/Materials & Methods section at the appropriate locations. Unless stated otherwise, results are presented as means ± s.d. P-values are reported in the appropriate location in the Results section for each experiment.

(For large datasets, or papers with a very large number of statistical tests, you may upload a single table file with tests, Ns, etc., with reference to sections in the manuscript.)

**Group allocation**

- Indicate how samples were allocated into experimental groups (in the case of clinical studies, please specify allocation to treatment method); if randomization was used, please also state if restricted randomization was applied
- Indicate if masking was used during group allocation, data collection and/or data analysis

Please outline where this information can be found within the submission (e.g., sections or figure legends), or explain why this information doesn’t apply to your submission:

Group allocation was only applicable to experiments comparing wildtype versus VR1-/- or VR2-/- mice, which were allocated based on genotype (only male mice 2-4months old were used for in vivo experiments). Information regarding this group allocation is provided in the Materials & Methods section.

**Additional data files (“source data”)**

- We encourage you to upload relevant additional data files, such as numerical data that are represented as a graph in a figure, or as a summary table
- Where provided, these should be in the most useful format, and they can be uploaded as “Source data” files linked to a main figure or table
- Include model definition files including the full list of parameters used
- Include code used for data analysis (e.g., R, MatLab)
- Avoid stating that data files are “available upon request”

Please indicate the figures or tables for which source data files have been provided:

Source data for each figure have been uploaded as ‘source data’ where appropriate. Sequencing datasets for STARR-seq and ATAC-seq have been deposited in the GEO database under accession numbers accession numbers GSE145257 (available from April 30, 2020) and GSE121464, respectively.
